# Do You Think I Am Living Well? A Four-Season Hair Cortisol Analysis on Leisure Horses in Different Housing and Management Conditions

**DOI:** 10.3390/ani11072141

**Published:** 2021-07-20

**Authors:** Silvia Michela Mazzola, Carla Colombani, Giulia Pizzamiglio, Simona Cannas, Clara Palestrini, Emanuela Dalla Costa, Alessia Libera Gazzonis, Arianna Bionda, Paola Crepaldi

**Affiliations:** 1 Dipartimento di Medicina Veterinaria, Università degli Studi di Milano, 26900 Lodi, Italy; giulia.pizza@hotmail.it (G.P.); simona.cannas@unimi.it (S.C.); clara.palestrini@unimi.it (C.P.); emanuela.dallacosta@unimi.it (E.D.C.); alessia.gazzonis@unimi.it (A.L.G.); 2 Dipartimento di Scienze Agrarie e Ambientali-Produzione, Territorio e Agroenergia, Università degli Studi di Milano, 20133 Milan, Italy; carla.colombani@unimi.it (C.C.); arianna.bionda@studenti.unimi.it (A.B.); paola.crepaldi@unimi.it (P.C.)

**Keywords:** horses management, welfare, paddock, natural horsemanship

## Abstract

**Simple Summary:**

The satisfaction of behavioral needs in leisure horses has begun to be considered a priority. There is growing awareness that horses kept in single boxes may be deprived of social contact and the possibility to perform natural behaviors. However, many practical factors may influence horses’ quality of life also in the paddock. In the literature, few studies have compared the effects of different variables related to paddock housing on leisure horses’ welfare. Therefore, managerial choices faced by owners, stables managers, and breeders cannot be based on concrete facts that are scientifically supported but only rely on experience, common sense, and anecdotal beliefs. This study aimed to assess and compare the chronic stress level in three groups of leisure horses, homogeneous in terms of sex and age, hosted in paddocks in structures in the same geographic area with a different daily routine, in order to verify which could be the one that best contributes to achieve the welfare of horses. The hair cortisol concentration, a reliable marker of chronic stress, was analyzed in 47 horses; samples were collected the same day for all the subjects four times during a year, once for each season. The results highlighted that the highest hair cortisol values were detected in the autumn and summer independently from the management strategies and were also significantly higher in individuals older than 15 years. The hair cortisol concentration was not influenced by horses’ sex or coat color. The comparison of the different management strategies showed that in the summer, autumn, and winter, the hair cortisol levels were significantly lower in horses who spent the night in the stables, indicating that those subjects had the best homeostasis. These findings, if confirmed by further studies, may be useful to enhance horse welfare and assist in management choice decision-making.

**Abstract:**

The satisfaction of leisure horses’ behavioral needs has begun to be considered a priority, linked to the awareness that horses kept in single boxes may be deprived of social contact and the possibility to perform natural behaviors. Several factors may influence horses’ quality of life also in the paddock, and there are very few data on the effects of those variables on leisure horses’ chronic stress, measurable in terms of activation of the hypothalamic–pituitary–adrenocortical axis. Therefore, managerial choices faced by owners and stables managers are only based on experience, common sense, and anecdotal beliefs. This study assessed and compared the chronic stress levels in leisure horses hosted in structures in the same geographic and climatic area with different daily routines to verify which management strategy could be the one that better contributes to achieving the welfare of horses. Forty-seven horses were divided into three groups homogeneous in terms of sex and age: Mixed management group (*n* = 12), Paddock group (*n* = 19), and Natural management group (*n* = 16). The hair cortisol concentration, a reliable marker of long-term stress, was analyzed in all the horses the same day at four time points of the year. In addition to management strategies, the influences of other variables (sex, age, coat color, and season) were evaluated. Independently from the management strategies, significantly higher hair cortisol values were detected in the autumn and summer, as well as in individuals older than 15 years. No significant differences were highlighted between the sexes or the coat colors. The comparison of the different management strategies showed that, in the summer, autumn, and winter, the hair cortisol levels were significantly lower in the Mixed management group horses than the Paddock group, highlighting that those subjects had better homeostasis. The Natural management group horses’ hair cortisol levels were intermediate between the other two groups of horses in all the seasons. Spending the night in the stables would seem to positively impact the well-being of the horses. These findings, if confirmed by further studies, may be helpful in enhancing horse welfare and assisting in managerial choice decision-making.

## 1. Introduction

Since domestication, the use of horses has profoundly varied: initially bred for meat or as a means of transport, they are now primarily grown for sport and leisure [1,2]. In the leisure horse industry, many people consider horses more as companion animals than as work animals [3], and in the last few years, fortunately, the satisfaction of horses’ behavioral needs has begun to be considered as one of the priorities. Even among horse owners, there is growing awareness that stabled horses may be deprived of opportunities both for social contacts and the possibility to perform natural behaviors, limited by stable designs and insufficient box dimensions [4]. In Northern Italy, this change of mentality has led to a modification in both the housing and management of horses, with the creation of structures very different from the traditional ones. Horses evolved as grazing and browsing herbivores [5], and it was demonstrated that free-ranging and pastured horses graze for most of their time (up to 18 h/day), foraging on a wide variety of species, selectively [4,5]. In this way, some stables have created large paddocks, variously equipped, where animals live in social groups for the whole day or only during daylight hours, depending on the management dynamics or on horse owners’ decisions. From an economic and managerial point of view, traditional stalling, which represents the most common form of equine housing in Europe and the USA [6], certainly has advantages, including a greater management efficiency, a faster and more accurate supervision of horses, and the possibility of hosting a large number of subjects in a single structure. Moreover, many owners and trainers believe that stabled horses are more protected from accidents that could happen in paddocks and during social interactions with other horses [7]. Conversely, stabled horses are dependent on humans for the selection, delivery, and timing of their diet, with restricted access to forage [6], the conditions very different from a free-ranging environment. This impact on the foraging behavior, associated with social restrictions, may affect horses’ welfare: horses spend less time eating and more time standing, a situation that has implications in horses’ physical and mental health [5].

Pasturing horses to improve their psychophysical well-being and prevent stabling-related problems is not without difficulties, because several factors, such as the group composition, can be tricky to optimize; in fact, it is rarely possible to mimic the composition of the natural harem bands of feral horses, which consists of one stallion and five to seven mares, yearlings, and foals [8,9]. In pastured horses, the composition of the groups in terms of the number, age, and sex of the members is determined by humans and may be frequently altered, consequently, due to the routines of owners, the changing dietary needs of the horse, and the availability of space; this social instability may lead to negative implications for the horses’ health [10]. Many other practical factors may significantly influence horses’ quality of life in the paddock; among these, dietary supplementation, the use of the blankets during cold periods, stabling during the night, the presence of insulated shelter, and structures designed to favor the activity and interactions of horses may be relevant.

Cortisol measurement is an indicator of hypothalamic-pituitary-adrenocortical axis (HPA) activity, which reflects the physiological responses to acute or long-term stress [11,12]. Plasma and saliva are point samples for the analysis of HPA activity; influenced by pulsatile circadian rhythmicity and environmental disturbances, plasma and saliva cortisol levels indicate a short retrospective timespan (from minutes to a few hours) [13,14]. The measurements of urine and feces, instead, generally assess HPA activity in 24 h or less [11,15]. This means that the assessment of chronic stress or long-term activity of the HPA would require elaborate sampling methods on these substrates [15,16]. Conversely, the hair cortisol concentration (HCC) is a marker of cortisol secretion and stress over long periods [17]. Systemic cortisol is incorporated into the hair via passive diffusion in the hair follicle from blood vessels during the active hair growth phase called anagen, forming retrospective fingerprints of cortisol concentrations. To properly use HCC as a biomarker of hypothalamic-pituitary-adrenocortical axis activity, the sampling method must ensure that specimens contain actively growing hairs. The “shave–reshave” technique requires that a specific area is shaved immediately before the beginning of the period of interest, and the regrown hair in the same area is reshaved at the end of this period [1,15]. HCC quantification is increasingly used in psycho-neuro-endocrinological studies in humans and, more recently, animal welfare research [15,17,18,19,20]. The sampling procedure, smooth and minimally invasive, and the extended time periods to which the data obtainable from a sample refer make the hair cortisol titration an extremely useful biomarker for the assessment of chronic stress in animals [15].

In the literature, as far as we know, only a few studies have compared the effects of different variables related to paddock housing on leisure horses’ chronic stress in terms of activation of the HPA [12]; moreover, little is published about how different management regimes affect horses’ health and welfare [1,21]. This means that managerial choices faced by owners, stables managers, and breeders cannot be based on scientifically supported facts but only on experience, common sense, and anecdotal beliefs.

In this study, therefore, the goal was to shed some light on this question: we assessed and compared the chronic stress levels in 47 leisure horses hosted in paddocks in structures in the same geographic area (south part of Garda Lake, north of Italy) with three different daily routines. We aimed to test the hypothesis that horses’ chronic stress levels could be related to different paddock housing strategies. For this purpose, we titrated hair cortisol in all the horses involved four times during the year, once for each season, having the foresight to sample hair on the same days and on the same body region through the shaving–reshaving method.

## 2. Materials and Methods

### 2.1. Stables

For this study, we selected three stables in the north of Italy, Lake Garda region, based on the different characteristics of horse management in the choices of horse owners. This region is classified as Cfa—a humid subtropical climate—following the Köppen–Geiger system. In April 2018, the average maximum and minimum temperatures were 18 and 10 °C, the rainfall was 8,5 mm (in 10 days of rain), and the average wind speed was 7.8 km per hour. The average pressure was 1015.7 Mb, the average humidity was 70%, the average cloud cover was 30%, and the sun hours were 322.15 in 14 sun days (https://www.worldweatheronline.com/desenzano-weather-averages/lombardia/it.aspx. Accessed on 5 May 2021). The choice to recruit three stables located in a very limited geographical areas was to ensure homogeneous climatic conditions for all the animals sampled, reducing the potential biases.

In Stable#1 (latitude: 45.445078, longitude: 10.755216), the management of stall-hosted show horses coexisted with vast paddock areas. Horse owners could choose whether to allow their horse to spend most of the day in paddock in social groups of about ten individuals; in this case, in the evening, horses were taken back to their individual boxes, where they received their ration of hay and feed and spent the night (Mixed management group). Alternatively, the horse owner may choose to permanently leave their horse in the paddocks, even during the night hours (Paddock group). Both the Paddock group and the Mixed management group horses received the same veterinary prophylaxis of stabled horses (anthelmintic and vaccines), and their health was carefully monitored by the staff and by the veterinarian who collaborated with the structures.

In Stable#2 (latitude: 45.447355, longitude: 10.538899), all horses’ management routines provided all-day access to the paddocks in social groups. As in Stable#1, horse owners could choose whether to let them back in the boxes during the night (Mixed management group) or leave them permanently in the paddock (Paddock group). All horses had a diet that included hay and feed, which were administered, depending on the group, in the paddock or in the boxes. Both the Paddock group and the Mixed management group horses received the same veterinary prophylaxis (anthelmintic and vaccines), and their health was carefully monitored by the staff and by the veterinarians who collaborated with the structure.

Stable#3 (latitude: 45.468592, longitude: 10.524228) was inspired by the principles of natural management, where horses lived in a herd free on 6 hectares of land, wood, and olive groves (Natural management group). Two natural ponds allowed horses to access water. There were three cement sheds, open on one side, which offered shelter to the animals, and four hay racks distributed to distant estate points to encourage the horses to move. Subjects that required additional concentrate feed wore a computer chip around their pastern that was read by the automatic oat dispenser to deliver the appropriate amount of concentrate daily. Horses were not subjected to any veterinary prophylaxis.

### 2.2. Animals

The study was planned to monitor horsehair cortisol concentrations during a one-year time lapse. Therefore, we initially recruited 66 subjects (22 for each stable), a number that was large enough to allow the analysis of the data even in the case that, for any reason, some horses were not available for the whole observation period. At the end of the sampling period, we limited the number of subjects to 47, since only these subjects did not change their housing conditions during the research period, and, for them, a veterinarian’s intervention was never required for actions that were not related to preventive treatments.

All horses were identified by reading the microchip number and using three photographs (two lateral and one frontal), which allowed recognition of the subjects throughout the observation period. All animals underwent a veterinary check-up to ensure that their health conditions were optimal and that no pathological conditions could interfere with the study data. Anamnestic history was collected for each horse. None of the horses enrolled in the study expressed stereotypic behaviors.

According to the type of housing, the animals were subdivided into three groups balanced in terms of sex, breeds, and working conditions (very light, maximum 1 h per day). The main characteristics of the horses enrolled and their division into groups are summarized in Table 1 and described in Table S1.

Group 1 horses (Mixed management group, *n* = 12, six from stable#1 and six from stable#2; Table 1 and Table S1) had access to the paddock during the day (from 8 to 18 in the spring–summer period and from 9 to 16 in the autumn–winter period, according to climate conditions), and they were led back to the stables in the evening hours, where they spent the night in individual boxes (4 × 4 m). Within the paddocks (both around 20,000 sqm in size; Figure S1), horses’ social groups were stable over the observation period, and the animals had free access to water (automatic concrete or metal drinkers) and shaded areas (trees and wooden huts). In both the stables, the boxes were inside insulated buildings made of brick and concrete, wooden and metal doors, and open windows that allowed visual contact, but not physical touch, with conspecifics. The bedding material was a natural product based on straw. Horses’ diets were calibrated according to individual needs and included, in addition to paddock grass and the hay placed in the racks, a supplement of concentrated feed administered in the morning and evening in the boxes. All animals were dewormed regularly through a periodic administration of anthelmintics and underwent vaccine prophylaxis. A farrier periodically took care of trimming the horses’ hooves; some horses were barefoot, while some horses were shod. No horse was clipped, and some were covered with blankets throughout the autumn–winter period.

Group 2 horses (Paddock group, *n* = 19, 11 from stable#1 and 8 from stable#2) lived in the paddock during day and night, independently from the weather conditions, in permanent social groups. The paddocks, both around 40,000 sqm in size (Figure S2), were structured with large, insulated sheds where horses could shelter during unfavorable weather conditions, hay racks, metal feeders, and automatic drinkers. Horses’ diets were calibrated according to individual needs and included, in addition to the paddock grass and the hay placed in the racks, a supplement of concentrated feed administered in the evening in separate feeders spaced along the perimeter of the paddock to minimize competition. All animals were dewormed regularly, through a periodic administration of anthelmintics, and underwent vaccine prophylaxis. A farrier regularly took care of trimming the horses’ hooves, which were all barefoot.

Group 3 horses (Natural management group, *n* = 16, all from stable#3) lived free in a herd in a wide area of permanent grassland (Figure S3) structured to evoke the wild environment with trees, natural water sources, and grasses. All horses who lived free in close contact with each other were managed with a holistic approach, according to the Natural Horsemanship philosophy [3], based on the horses’ natural instincts and methods of communication and were without any medical treatment, including deworming and vaccinations. None of the horses wore a blanket in any season, and all were barefoot, without any regular trimming.

### 2.3. Sample Collection

All methods and procedures used in this study followed the guidelines of the Italian law for the care and use of animals (D.L. 4 March 2014 n. 26) and EU directive (2010/63/EU). All the owners were informed in detail of the objectives and methods of the research and consented to participation by issuing a full informed consent.

The data discussed here were collected from April 2018 to January 2019. The research protocol was structured on four analysis points, which occurred during the third week of April, July, October 2018, and January 2019. Hair samples were collected from each horse by shaving 2 square cm of the neck region to the level of the skin at the same point of each sampling time (shaving and reshaving method). Clean and dry hair samples were stored in polypropylene tubes (Falcon, Fisher Scientific, Rodano, Italy) at room temperature until analysis.

### 2.4. Laboratory Procedure

Hair cortisol extraction was performed following the procedure described by Burnett et al. [22,23] and Banse et al. [18]. One hundred milligrams of hair samples from each horses’ time point were washed twice with double-distilled water, and the sample was allowed to dry overnight. Hair samples were washed with isopropanol for 3 min and allowed to dry completely for 12 h. The washed, dried hair was powdered in a ball mill (Retsch MM400, Retsch-Allee 1–5, Haan, Germany), and 40 mg of shredded hair was weighed into vials; then, 2 mL of 99.9% methanol (Sigma-Aldrich, viale Monte Rosa 93, Milano, Italy) was added. The vials were tightly capped and sonicated for 30 min (Branson 2510, Branson Ultrasonic Corp., Danbury, CT, USA). The samples were then incubated for 12 h in an orbital linear shaking water bath (Grant OLS200, Fisher Scientific, Loughborough, LE11 5RG, Loughborough, UK) at 100 rpm and 50 °C to extract the steroids, and then, 1.5 mL of the supernatant was pipetted into a 2.5-mL microcentrifuge tube and evaporated at 45 °C under a stream of ultrapure nitrogen gas. The samples were reconstituted in 200 μL of phosphate-buffered saline (Merk Millipore, Milano, Italy).

Hair cortisol was analyzed using a commercially available assay kit designed to accurately measure the cortisol levels in a variety of sample matrices (Enzo Life Sciences, Farmingdale, NY, USA). Samples were aliquoted into wells in duplicate (100 μL), and absorbance was measured using a wavelength of 405 nm in a microplate reader (Multiskan EX, LabSystem, Thermo Fisher Scientific, Milan, Italy). The mean recovery was 108.9% ± 8.3%, while the average intra- and inter-assay coefficients of variation were 3.9% and 7.7%, respectively. The assay sensitivity was 56.72 pg/mL (range 156–10,000 pg/mL). The laboratory researcher was blinded to the hypotheses and conditions.

### 2.5. Statistical Analysis

Statistical analysis was performed with JMP statistical discovery software (SAS, Cary, NC, USA) using the following mixed model:

Y_ijkl_ = M_I_ + G_J_ + S_K_ +A_L_ + (M∗S)_I∗K_ + (M∗A)_I∗L_ + (S∗A)_K∗L_ + (H)_m_ + e_IJKL_

where:

- Y_ijkl_ = cortisol level (ng/mL);
- fixed effects:
  ○ management (M) with three levels (I = mixed, paddock, and natural);
  ○ sex (G) with two levels (J = female and male);
  ○ season (S) with four levels (K = Spring, Summer, Autumn, and Winter);
  ○ age group (A) with two levels (L = under 15 years old or ≥ 15)
- random effect:
  ○ horses (H) with m = horse identification number;
- e_IJKL_ = residual component.

On the significant factors, the Restricted Maximum Likelihood (REML) Variance Component Estimates test was used.

One-way ANOVA was used to compare the HCC in different conditions: coat color, use or not use of a blanket, and season; results were confirmed with a Welch’s test when appropriate. To reduce data dispersion, horses’ ages were grouped into two categories (younger and older than 15 years), and coat color was grouped into three categories by the genetic colors’ origin [24]: bay–black, chestnut, and diluted and piebald (which included gray, roan, and piebald).

## 3. Results

The HCC values, grouped by the main variables considered, are shown in Table 2.

The analysis of the horsehair cortisol values obtained in all seasons in the two stables that housed the Mixed management and Paddock group animals evidenced no statistically significant differences among inter-group individuals (*p* = 0.19), demonstrating the homogeneity of the subjects within the groups.

In the factorial mixed model, the following fixed factors resulted as significantly associated with HCC: management (*p* < 0.001), age group (*p* = 0.02), and season (*p* = 0.049). On the contrary, there was no statistically significant effect of sex (*p* = 0.42) and the interaction between the season and the age of the horses (*p* = 0.46), between housing management and the age (*p* = 0.37), and between housing and season (*p* = 0.23). The R-Square of the model was 0.52, meaning that more than half of the variability of the HCC depended on the included factors. The mixed model showed appropriate degrees of freedom to evaluate the interactions between the parameters considered.

**Figure 1:**
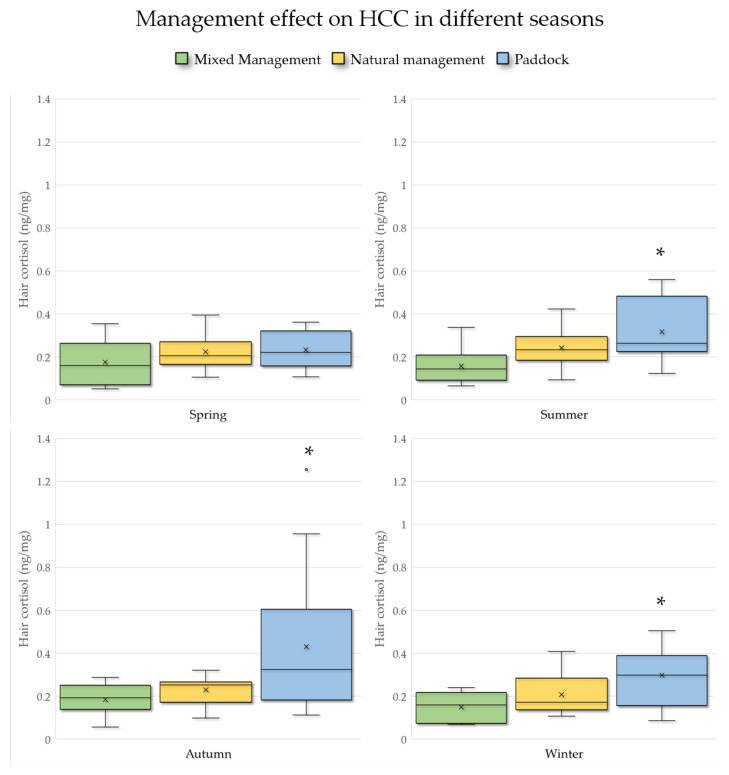
Box plots of the horsehair cortisol (HCC) levels in the different management conditions, in each season, for all the included horses (*n* = 47). * = significantly higher values. *p* ≤ 0.05 was considered statistically significant.

All the factors included in the model were further investigated. The results showed that the management strategies the horses underwent induced a highly significant effect on the HCC. The post-hoc test highlighted that the Paddock group was associated with significantly higher hair cortisol values than the other two, with a least-square mean ± SE of 0.33 ng/mg ± 0.03, while the Mixed management (0.17 ng/mg ± 0.01) and Natural management (0.23 ng/mg ± 0.01) groups did not differ. The Natural management group had an intermediate HCC value compared to the other two groups. The interaction between house management and season was not significant in the model, but when analyzing with ANOVA only the HCC data obtained in the three groups of horses, separately in the different seasons (Figure 1), it can be noticed that, during the springtime, the management conditions did not induce a statistically significant difference in the hair cortisol levels (*p* = 0.31). On the contrary, in the summer (*p* = 0.002), autumn (*p* = 0.009), and winter (*p* = 0.012), the management strategies evoked a different hypothalamic–pituitary–adrenocortical axis activation, evidenced by statistically higher HCC levels in the Paddock group, as well as a greater variability, especially during the autumn.

In the model, the effect of age class (<15 years and ≥15 years) on the HCC was statistically significant; the data highlighted that hair cortisol had a higher least square mean in individuals older than 15 years (0.30 ng/mg ± 0.02) compared with the younger ones (0.22 ng/mg ± 0.02). The interaction between the season and age was not significant and, in fact, the same trend was found in all the seasons.

**Figure 2:**
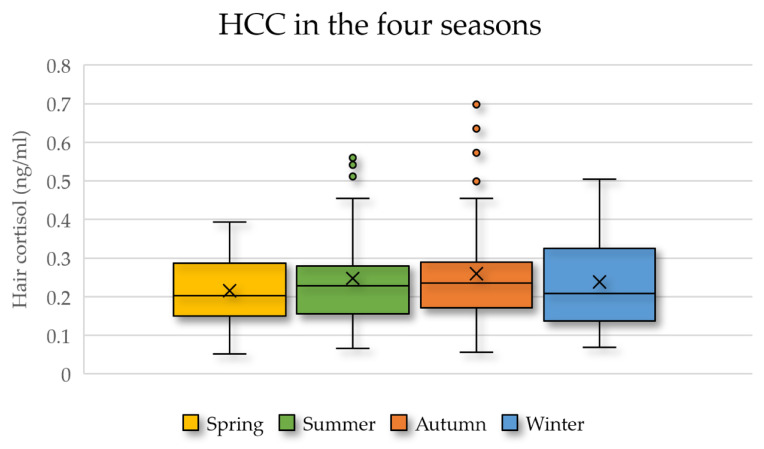
Box plot of the hair cortisol levels during the different seasons for all the included horses (*n* = 47).

The season, considered as a factor in the statistical model, showed a significance just below the statistical threshold of 5%, but the post-hoc test did not show significant differences between the seasons; the autumn had the highest least square mean ± SE, equal to 0.29 ng/mg ± 0.02, followed by the summer (0.26 ng/mg ± 0.02), winter (0.22 ng/mg ± 0.03), and spring (0.22 ng/mg ± 0.02). When the HCC was analyzed in relation to the different seasons only (ANOVA), no differences were found, but the winter showed a greater variability in terms of the interquartile range, whereas, in the autumn, there was the largest number of outliers (Figure 2).

The association of the HCC with two more variables, not included in the model, was analyzed. The ANOVA showed that the coat color did not significantly affect the HCC (*p* = 0.93).

Since some horses that belonged to the Mixed management group were covered with a blanket in the winter and autumn, we evaluated if this practice could influence their HCC in comparison with other horses of the same group. The variance analysis among the individuals with (*n* = 6, mean ± SE = 0.21 ng/mg ± 0.02) or without the coverage (*n* = 6, 0.11 ng/mg ± 0.02) evidenced statistically significant lower HCC in horses without a blanket (*p* = 0.0008).

## 4. Discussion

Horses are one of the most popular leisure companion animals. Italy counted 367,561 horses in 2017 [25], reared in 154,948 farms [26] and managed mainly with traditional husbandry systems, with the horses kept confined and single boxes. Despite the growing consciousness that these systems, considered optimal to prevent injuries and allow individual monitoring, often ignore horses’ basic needs such as social contacts, foraging, and locomotion, there is no data about how different management regimes affect the health and welfare of horses. Welfare is closely connected to management and housing practices, since it refers to the individuals’ state and coping responses to the environment. Animal welfare is a multidimensional concept, which can be measured through physiological, behavioral, and immunological animal-based indicators [27].

Cortisol accumulates in the hair over time; therefore, an HCC analysis offers a noninvasive and reliable method to assess long-term systemic cortisol production, reflecting the hypothalamic–pituitary–adrenocortical axis activation [19]. The present study aimed to analyze the effects of management strategies on hair cortisol concentrations in leisure horses living all year-round in social groups in large paddocks, with (Mixed management group) and without (Paddock group) returning overnight to their boxes or following the natural horsemanship approach [3] (Natural management group).

In the statistical model presented here, we considered the effects of management and those of season, age, and sex, on the chronic stress of 47 horses while keeping into account the individual variability of HCC. Management emerged as the most significant factor affecting horses’ HCC. Particularly, the subjects belonging to the Paddock group showed higher HCC levels, whereas the lowest were found in the Mixed management group. The management differences between these groups are linked to the overnight accommodations; in both the stables involved in the study, the horses of the Mixed management group were brought back to the stable in the evening at a time that varied with the seasons’ daylight hours; in contrast, the Paddock group horses spent the night outside, like those of the Natural management group. Spending the night in the stables would seem to impact the well-being of the horses positively, and this could be related, at least in part, to the sleep quality [28]; sleep is an essential facilitator of physical well-being and optimal mental functioning, especially when we consider that horses, being large prey animals, sleep for an average of only 3 h a day [29]. Moreover, the average duration of nocturnal recumbent behavior, a recognized marker for horses’ most effective rest [30], was found to be positively correlated to competition performance [31]. The current knowledge identifies some factors that may alter equine sleep and, thus, their welfare, and the influence of equine husbandry practices on nocturnal behavioral profiles should not be underestimated. For example, the bedding material and its cushioning properties are very important for horses; it has been shown that straw bedding in the stable promotes biologically significant behaviors, including recumbent action [32]. In our study, the structures of both stables involved were conceived with a large box designed to enable social interactions, with straw bedding, well-aerated and thermally insulated, and shown to ameliorate the welfare conditions of the horses who spent the night there. Our results demonstrated that, for horses that spent the day grazing, with social interaction and free to move, stabling could represent a nocturnal environment that promotes sleep. On the other hand, the factors that could negatively influence the well-being of the horses spending the night in the paddock, inducing a greater activation of the hypothalamic–pituitary–adrenal axis, could be multiple. More likely, they could be a series of concomitant factors, such as the weather, ambient temperature, insects, or interactions within other horses, which, depending on the season and on the horses’ perception, might interfere with the horses’ homeostasis [33]. All groups of horses exhibited stable social dynamics; horse handlers and owners did not report observations of episodes of aggression in any groups, not even aggressive interactions induced by competition for resources. It should be noted that the management of the horses in our study excluded known stressors that might affect cortisol release, such as transport [34], change of stable or group mates [35], equestrian exercise [4,36], or veterinary procedures [37]. The present study results also highlighted that the Natural management group horses’ hair cortisol levels were intermediate between the other two groups. This housing model is close, as far as possible, to what is present in natural, pasture-like conditions, where free-range horses have a structured social environment that allows horses to also follow a natural circadian rhythm of behavioral patterns. Although the weather conditions, superimposable in all the groups, could not have influenced the results, other environmental variables may have been different for these horses and, thus, deserve further investigation: nutrition, trainer experience, medical treatments, regular trimming, and degree of handling, seen as departing radically from traditional approaches [3].

In the present study, the effect of the management on the HCC was more pronounced in the summer, autumn, and winter, whereas, in the spring, the cortisol levels were almost the same in all the three groups (Figure 1). The lack of differences in the spring could be related to the mild climate of this season in the particular area of Northern Italy, the south part of Garda Lake, where the horses were sampled; in these favorable conditions, an overnight stay in the stables becomes less critical. Nevertheless, it should be emphasized that the climatic conditions throughout the year never differed from those defined in the zone of least thermoregulatory effort, which describes an environmental temperature span under which the animal is not under thermal stress and neither needs to mobilize energy reserves nor to pant or sweat to keep a steady core body temperature [38].

This particular climate could also explain why the differences in the HCC were minimal in the different seasons, with slightly higher values detected in the autumn and summer (Figure 2). Higher autumnal HCC are in agreement with the described pituitary gland circannual rhythm of the equine, which has the highest ACTH output in the fall [18,39,40,41], and the lowest spring HCC levels were consistent with the observations of Aurich et al. [11], who reported lower salivary cortisol levels in March and April when considering the period from December to May. Further studies involving animals living in different climatic conditions may shed further light on the influence of external environment on horses’ welfare.

The mild external temperatures recorded in the autumn and winter may have influenced our findings concerning the hair cortisol levels of horses using blankets. In both stables involved in the study, some of the Mixed management horses wore a blanket continuously during the years’ coldest months. Although the number of subjects was limited, the statistical analysis revealed that those without blankets had statistically significant lower HCC levels. This is in accordance with the study of Mejdell et al., which described, in a Nordic country, a method in which horses learned to communicate to the handler whether they wanted to have a blanket on or not [42]; by comparing horses’ choices at days with different weather conditions, they highlighted that the horses’ indications strongly represented individual preferences and were dependent on the thermoregulatory challenge related to several climatic factors, such as ambient temperature, wind, and precipitation on the test days. Therefore, it is possible that, in the present study, the horses of the Mixed management group, not being exposed to particularly demanding thermoregulatory challenges, experienced the blanket more as a limitation in movement than a thermal benefit [43]. In the horse, the withers’ dorsal spinous processes are particularly vulnerable to pressure, and even though the modern horse blanket materials and insulation are lightweight and inflict a relatively small amount of pressure, some pressure sores may develop when applied for extended periods [43]. However, it should be emphasized that none of the horses of our study showed visible lesions attributable to blanket use.

The present study also highlighted that sex and coat color did not significantly influenced the HCC, whereas age did; individuals older than 15 years were associated with significantly higher HCC than younger ones. An increase in hair cortisol related to age was also described in humans by Feller et al. [44], while, contrary to the findings of the present study, Hart et al. evidenced a lack of statistical significance of an effect of age on serum cortisol of 57 healthy horses aged 1–26 years [33]. The difference we found could be related to the lower ability of elderly subjects to comply with stressors, which is why older horses should be even more carefully managed.

## 5. Conclusions

The awareness that leisure horses’ welfare is related to the satisfaction of their behavioral needs makes it increasingly necessary to investigate the question from a scientific perspective; at present, due to the lack of scientific literature about the effect of management on horses’ welfare, breeders, stables managers, and owners can only rely on experience and common sense. This is the first study that has shed light on the relevance that some management variables, so far scarcely considered, could have on horses’ homeostasis using the hair cortisol concentration, a reliable and noninvasive indicator of chronic stress in the species. The findings presented here could help the efforts made to ensure that management decisions will lead to an improvement in horses’ living conditions.

## Figures and Tables

**Table 1:** Scheme of the horses involved in the study and group characteristics.

Stable	Horses (Number)	Group	Horses (Number)	Age (Mean ± SD)	Female (Number)	Gelding Male (Number)	Coat Color (Number)
# 1	17	Mixed management	6	13.8 ± 3.3	2	4	diluted/piebald = 1 bay–black = 3 chestnut = 2
		Paddock	11	14.2 ± 6.3	6	5	diluted/piebald = 2 bay–black = 5 chestnut = 4
# 2	14	Mixed management	6	9.8 ± 5.7	3	3	diluted/piebald = 1 bay–black = 4 chestnut = 1
		Paddock	8	8.6 ± 5.4	5	3	diluted/piebald = 2 bay–black = 5 chestnut = 1
# 3	16	Natural Management	16	17.7 ± 6.4	5	11	diluted/piebald = 6 bay–black = 4 chestnut = 6

**Table 2:** Hair cortisol mean values, grouped by season, age, sex, and coat color in different managements. SE = standard error of the mean. * = Significantly higher hair cortisol when different managements are compared.

Variable			Hair Cortisol (ng/mg, Mean ± SE)			
			Mixed Management	Natural Management	Paddock	Overall Mean
Season	Spring	*n* = 39	0.18 ± 0.03	0.22 ± 0.02	0.23 ± 0.02	0.21 ± 0.01
	Summer	*n* = 46	0.16 ± 0.02	0.24 ± 0.02	0.35 ± 0.05 *	0.26 ± 0.02
	Autumn	*n* = 40	0.18 ± 0.02	0.23 ± 0.02	0.43 ± 0.08 *	0.3 ± 0.04
	Winter	*n* = 37	0.15 ± 0.03	0.21 ± 0.03	0.30 ± 0.03 *	0.24 ± 0.02
Age	<15 years old	*n* = 27	0.14 ± 0.01	0.22 ± 0.02	0.28 ± 0.03	0.22 ± 0.02
	≥15 years old	*n* = 20	0.23 ± 0.02	0.23 ± 0.01	0.39 ± 0.04	0.3 ± 0.02
Sex	Female	*n* = 23	0.18 ± 0.02	0.27 ± 0.3	0.33 ± 0.04	0.29 ± 0.02
	Gelding male	*n* = 24	0.16 ± 0.02	0.21 ± 0.01	0.33 ± 0.04	0.23 ± 0.01
Coat color	Bay-black	*n* = 21	0.16 ± 0.02	0.20 ± 0.03	0.33 ± 0.04	0.25 ± 0.02
	Diluted-piebald	*n* = 12	0.16 ± 0.02	0.25 ± 0.02	0.31 ± 0.05	0.25 ± 0.02
	Chestnut	*n* = 14	0.20 ± 0.03	0.22 ± 0.02	0.34 ± 0.04	0.26 ± 0.02

## Data Availability

The data generated and analyzed during this study are included in this article.

## Supplementary Materials

The following are available online at https://www.mdpi.com/article/10.3390/ani11072141/s1: Figure S1: Mixed management group paddock areas. Figure S2: Paddock areas of the Paddock management group. Figure S3: Paddock area of the Natural management group. **Table S1**: Main characteristics of horses enrolled, their stables and their division into groups.
