# Supplementary material for: Do You Think I Am Living Well? A Four-Season Hair Cortisol Analysis on Leisure Horses in Different Housing and Management Conditions
Source: Animals (Basel). 2021 Jul 20;11(7):2141. doi: 10.3390/ani11072141 (PMC8300697; doi:10.3390/ani11072141)
Supplement: Supplementary file 1 [file animals-11-02141-s001.zip › animals-1261141-supplementary.pdf]

Horse #	Stable #	Group	Age	Gender	Breed	Coat color	blanket
1	1	Mixed management	15	M	paint horse	piebald (b/w)	yes
2	1	Mixed management	11	F	quarter horse	dark bay	yes
3	1	Mixed management	9	M	sella italiano	bay	no
4	1	Mixed management	16	M	sella italiano	bay	no
5	1	Mixed management	18	F	dutch warmblood	chestnut	yes
6	1	Mixed management	14	M	selle français	chestnut	yes
7	2	Mixed management	14	F	sella italiano	bay	yes
8	2	Mixed management	14	F	sella italiano	gray	yes
9	2	Mixed management	1,5	M	sella italiano	bay	no
10	2	Mixed management	1,5	M	trotter	chestnut	no
11	2	Mixed management	1,5	M	trotter	black	no
12	2	Mixed management	1,5	M	trotter	bay	no
13	3	Natural management	14	M	paint horse	piebald (b/w)	no
14	3	Natural management	14	F	thoroughbred	dark bay	no
15	3	Natural management	21	F	quarter horse	chestnut	no
16	3	Natural management	12	M	maremmano	dark bay	no
17	3	Natural management	14	F	thoroughbred	gray	no
18	3	Natural management	9	M	spagnolo	chestnut	no
19	3	Natural management	17	M	paint horse	piebald (b/w)	no
20	3	Natural management	21	M	sella italiano	chestnut	no
21	3	Natural management	25	F	quarter/trotter	bay	no
22	3	Natural management	30	M	argentino	roan	no
23	3	Natural management	20	F	arabian horse	gray	no
24	3	Natural management	22	M	sella italiano	chestnut	no
25	3	Natural management	25	M	thoroughbred	chestnut	no
26	3	Natural management	25	F	selle français	bay	no
27	3	Natural management	10	M	quarter horse	chestnut	no
28	3	Natural management	8	F	thoroughbred	gray	no
29	1	Paddock	22	F	hanoverian horse	bay	no
30	1	Paddock	3	F	sella italiano	bay	no
31	1	Paddock	21	M	thoroughbred	gray	no
32	1	Paddock	3	F	selle français	black	no
33	1	Paddock	3	M	selle français	bay	no
34	1	Paddock	3	F	sella italiano	chestnut	no
35	1	Paddock	21	M	thoroughbred	gray	no
36	1	Paddock	15	F	dutch warmblood	chestnut	no
37	1	Paddock	3	M	thoroughbred	black	no
38	1	Paddock	12	F	selle français	chestnut	no
39	1	Paddock	18	M	hanoverian horse	chestnut	no

40	2	Paddock	3	F	trotter	bay	no
41	2	Paddock	8	M	trotter	chestnut	no
42	2	Paddock	6	M	paint horse	piebald (c/w)	no
43	2	Paddock	8	F	paint horse	piebald (c/w)	no
44	2	Paddock	18	F	trotter	bay	no
45	2	Paddock	13	F	trotter	bay	no
46	2	Paddock	5	M	sella italiano	bay	yes
47	2	Paddock	18	F	sella italiano	bay	no

Table S1. Main characteristics of horses enrolled, their stables and their division into groups.

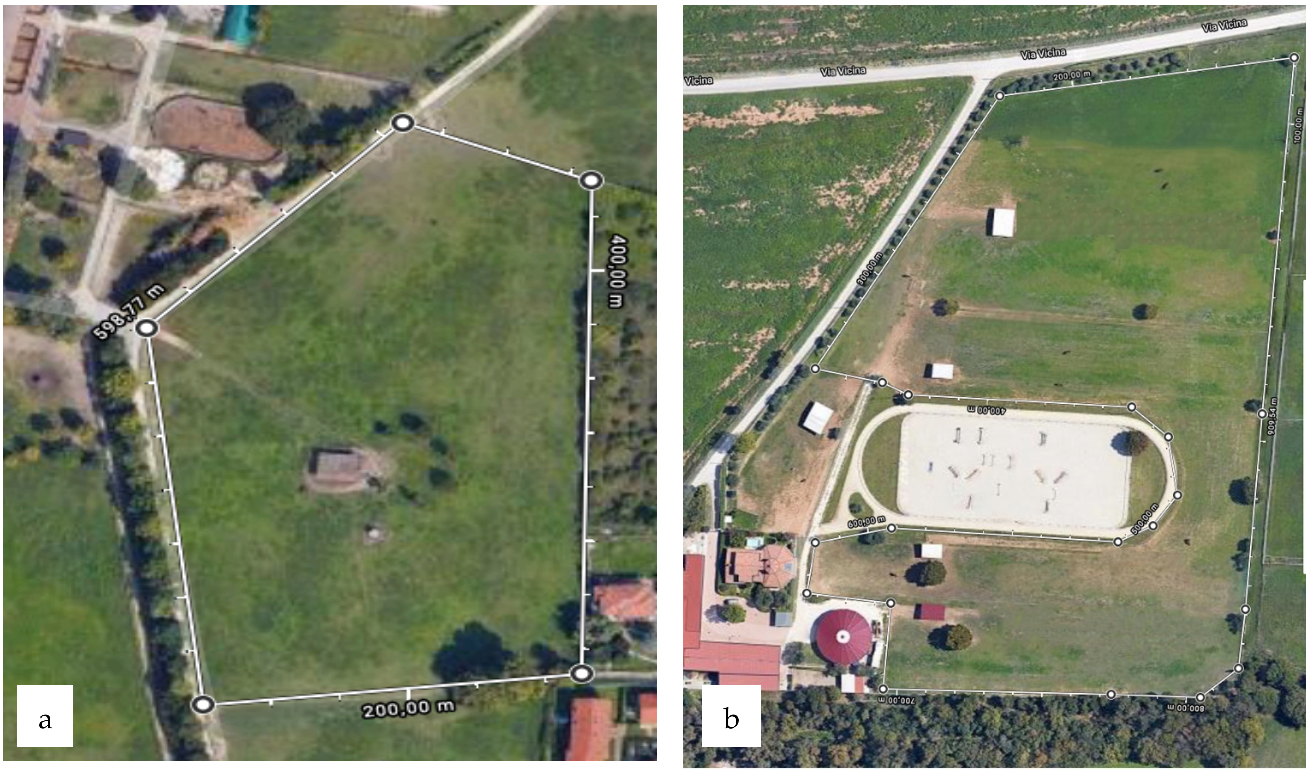

Figure S1. *Mixed management group* paddock areas. In **a**: paddock of Stable 1 (22.763,09 m^2); in **b**: paddock of Stable 2 (22.841,80 m).

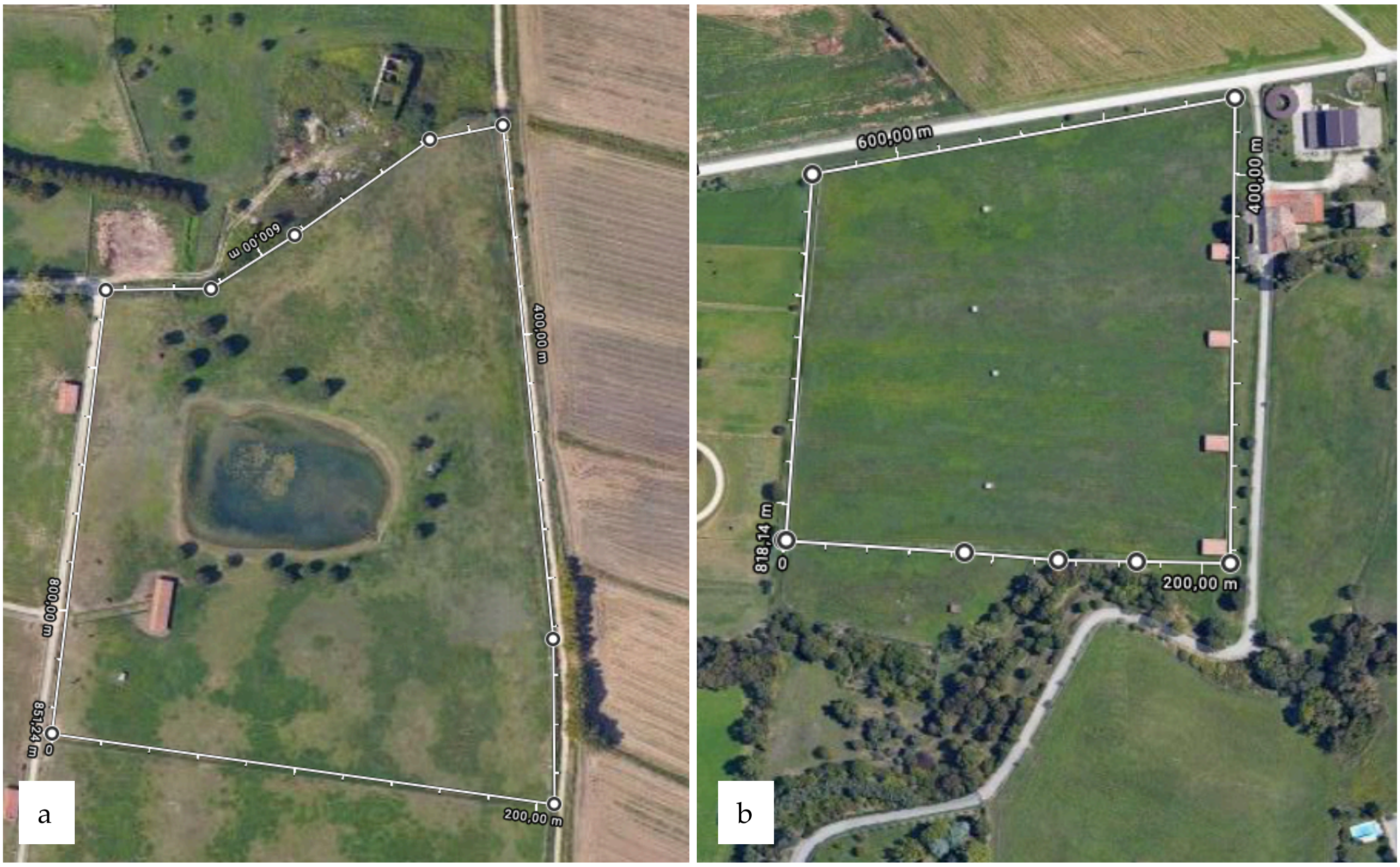

Figure S2. Paddock areas of the *Paddock group*. In **a**: paddock of Stable 1 (42.799,97 m²); in **b**: paddock of Stable 2 (45.582,59 m²).

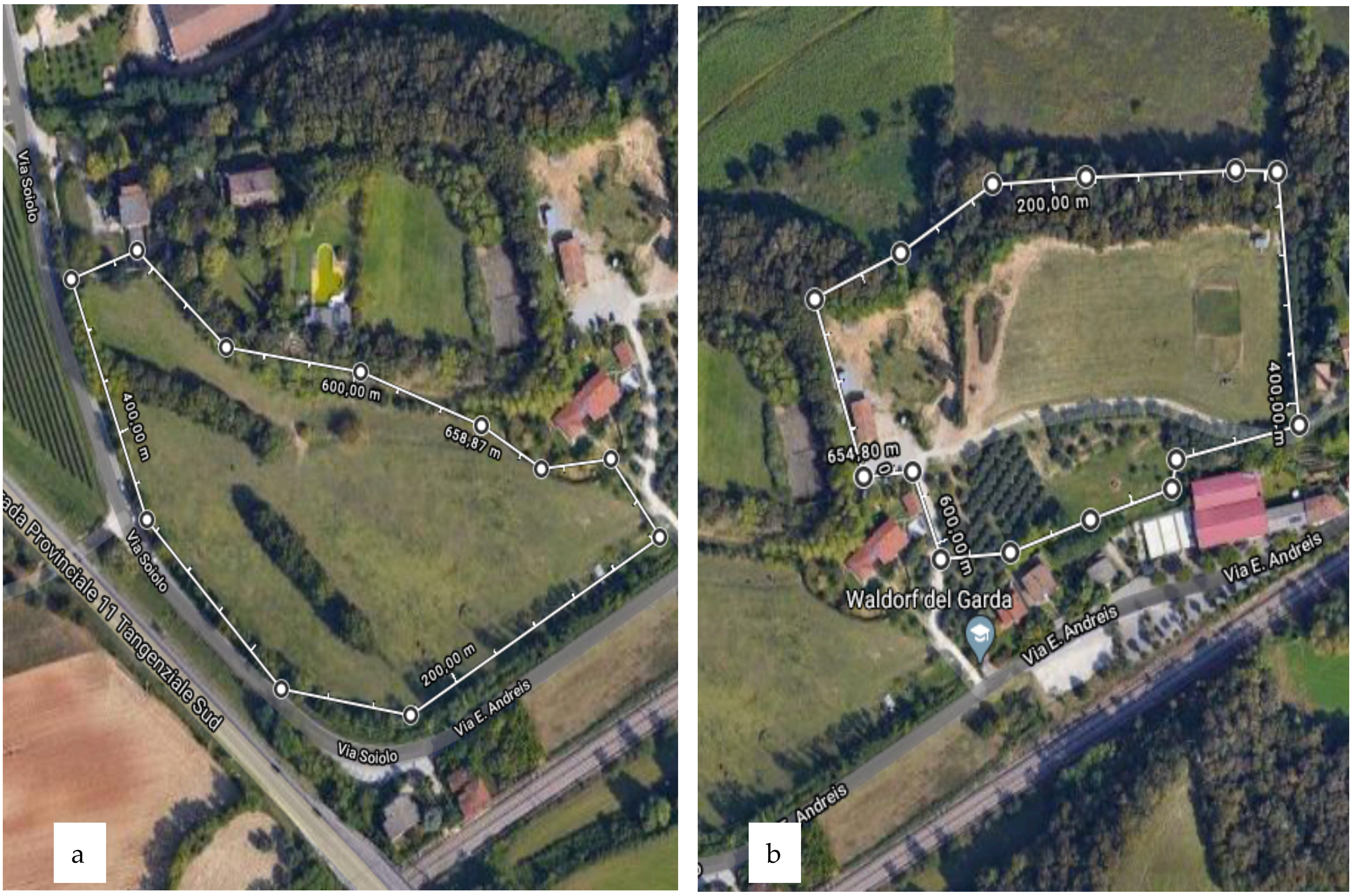

Figure S3. Paddock area of the *Natural management* group, both in Stable 3. Horses were free to move from one paddock (**a**; 21.479,86 m²) to the other (**b**; 25.783,04 m²) through a gate, which could be closed if deemed necessary.
